# Identification and characterization of a novel broad-spectrum antifungal compound targeting the *Aspergillus fumigatus* cell wall and cell membrane and inducing oxidative stress

**DOI:** 10.1128/spectrum.03143-25

**Published:** 2026-01-28

**Authors:** Ranying He, Jin Xie, Jinbin Hao, Bin Wang, Cheng Jin, Wenxia Fang

**Affiliations:** 1 Institute of Biological Sciences and Technology, Guangxi Academy of Sciences 245477, Nanning, Guangxi, China; 2 College of Life Science and Technology, Guangxi University 622308, Nanning, Guangxi, China; 3 State Key Laboratory of Microbial Diversity and Innovative Utilization, Institute of Microbiology, Chinese Academy of Sciences 85387, Beijing, China; University of Texas Medical Branch at Galveston, Galveston, Texas, USA

**Keywords:** natural products, antifungal, *Aspergillus fumigatus*, *Kitasatospora melanogena*, cell wall and cell membrane target, oxidative stress

## Abstract

The substantial increase in fungal diseases, the limited availability of antifungal drugs, and rising resistance to existing treatments highlight the urgent need for developing novel antifungal drugs. Natural products, known for their diverse bioactivities and unique structures, remain critical sources for antifungal drug discovery. In this study, we aimed to identify novel antifungal compounds from soil microbes and isolated a novel 28-membered polyene macrolide, compound **1**, from *Kitasatospora melanogena*. This compound demonstrated potent antifungal activity, with MICs of 4–16 μg/mL against *Aspergillus fumigatus*, *Aspergillus flavus*, *Candida albicans*, and *Cryptococcus neoformans*. Treatment with compound **1** led to significant growth reduction and disruption of the cell wall and membrane in *A. fumigatus* compared to the wild-type. To understand the underlying mechanism, we performed a transcriptomic analysis of *A. fumigatus* exposed to compound **1**, revealing a substantial downregulation of genes involved in cell wall and membrane biogenesis, as well as those encoding integral membrane proteins and multidrug transporters. Additionally, compound **1** induced oxidative stress, leading to elevated reactive oxygen species levels. These multifaceted mechanisms highlight the potential of *K. melanogena* natural products in the development of novel antifungal agents.

**IMPORTANCE:**

Fungal infections cause substantial morbidity and mortality worldwide, yet current treatments are limited and increasingly undermined by resistance. Natural products remain a proven source of antifungal agents, but few new scaffolds have been introduced in recent decades. We identified a novel polyene macrolide from *Kitasatospora melanogena* with potent activity against major fungal pathogens. This compound disrupts both the fungal cell wall and membrane while inducing oxidative stress, revealing a multifaceted mechanism of action. Our findings highlight soil microbes as valuable reservoirs for antifungal discovery and provide a promising lead for the development of next-generation therapies against life-threatening fungal diseases.

## INTRODUCTION

*Aspergillus fumigatus* is a widespread saprophytic filamentous fungus with a high sporulation capacity, making it one of the most prevalent airborne fungal pathogens in natural environments (1). Recognizing the global burden of fungal diseases, the World Health Organization (WHO) published the first-ever fungal priority pathogen list in late 2022, placing *A. fumigatus* in the critical priority group (2). *A. fumigatus* is responsible for claiming hundreds of thousands of lives annually and is responsible for a range of diseases, with invasive aspergillosis (IA) being the most common and severe, characterized by a high mortality rate (3, 4). IA affects approximately 2.1 million people globally each year, particularly in the context of chronic obstructive pulmonary disease, intensive care, lung cancer, or hematological malignancies, resulting in over 1.8 million deaths annually (5). Despite significant human health concerns, less than 1.5% of all research funding for infectious diseases is allocated to fungal pathogens. Consequently, the antifungal pharmacopeia is limited to only four classes—triazoles, polyenes, echinocandins, and pyrimidine analog flucytosine—used in clinical practice, with few new medications under development (6, 7). The limited pharmacopeia is further exacerbated by emerging antifungal drug resistance in pathogenic species (8), making the development of novel antifungal drugs imperative. Natural products, which are diverse chemical entities originating from bacteria, fungi, plants, and animals, have long served as a valuable source of compounds for drug discovery (9, 10). They offer several advantages, including lower costs, easier accessibility, fewer adverse reactions, and a reduced negative impact on human health (11, 12). For instance, natural products like cinnamaldehyde and eugenol have been reported to be effective natural fungicides, biocontrol agents, and non-toxic biopreservatives. They display significant antifungal activity against *A. flavus* (13).

In this study, our objective was to identify natural antifungals. From soil samples collected in Banlao, Yunnan Province, we isolated an actinomycete strain named BL11. This strain demonstrated broad-spectrum antifungal activity. Phylogenetic analysis revealed that strain BL11 belongs to the genus *Kitasatospora*. It is grouped in the same clade as *K. melanogena*, with a bootstrap support value of 87%. Employing liquid chromatography-mass spectrometry and nuclear magnetic resonance (NMR), we isolated a novel compound from strain BL11, which was designated as compound **1**. We then evaluated the inhibitory effect of compound **1** against various pathogenic fungi. For the first time, we also assessed its growth inhibition effect on *A. fumigatus*, along with exploring its potential underlying mechanism of action.

## RESULTS

### Initial screening and identification of strains

**Fig 1:**
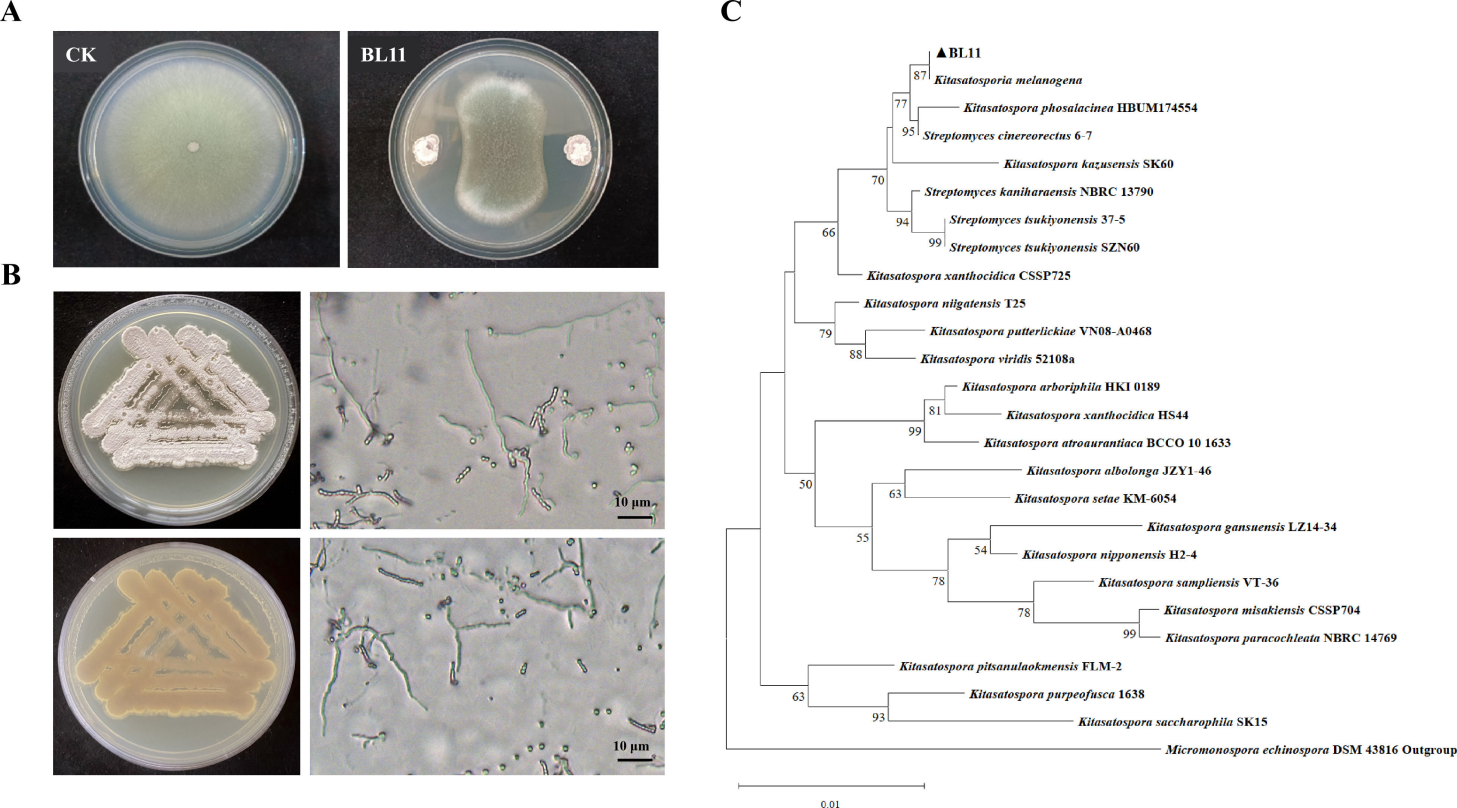
Screening and identification of antifungal strain BL11. (**A**) Antagonistic activity of strain BL11 against *A. fumigatus*, demonstrating significant growth inhibition. (**B**) Morphological characterization of strain BL11. Colonies were cultured on YAG medium, and mycelial morphology was visualized using a Leica DM6B fluorescence microscope (scale bar is 10 µm). (**C**) Phylogenetic analysis of strain BL11 based on the 16S rRNA gene sequence. The phylogenetic tree was constructed with 1,000 bootstrap replicates; bootstrap values are indicated at the nodes. The scale bar denotes sequence divergence.

A total of 77 actinomycete strains were isolated from soil samples. These strains were then screened for their antifungal activity against *A. fumigatus*. Among them, strain BL11 exhibited the strongest inhibitory effect (Fig. 1A). The morphological characteristics of BL11 were then examined in detail. The strain formed robust colonies with a dry, wrinkled surface. The colony underside displayed a light brown pigmentation, and abundant white aerial mycelia were present. Microscopic observations further revealed thin, short hyphae and chains of oval-shaped aerial spores, each approximately 2 μm in length (Fig. 1B).

In terms of taxonomic classification, an approximately 1,468 bp 16S rRNA gene fragment was successfully amplified from strain BL11. Based on the analysis of the 16S rRNA gene sequence, a phylogenetic tree was constructed. When compared to the outgroup, all the members of the genus *Kitasatospora* were clustered together in a single group. Strain BL11 was classified within the same clade as *K. melanogena*, and this classification was supported by a bootstrap value of 87% (Fig. 1C).

### Extraction, purification, and structural identification of compound 1

**Fig 2:**
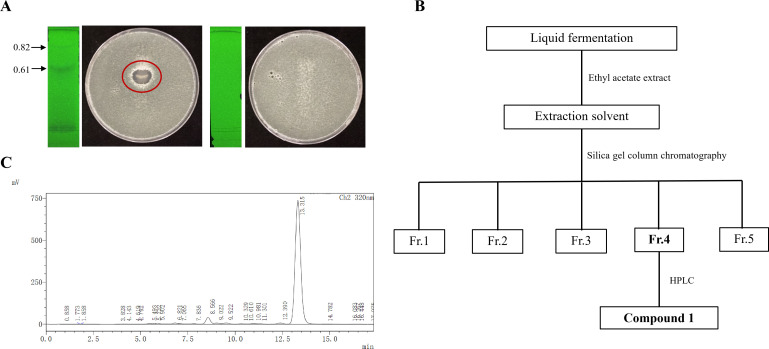
Isolation and purification of compound 1. (**A**) Bioautography assay of BL11 extracts. The chromatogram was visualized under UV light at 254 nm using a mobile phase of CH₂Cl₂:MeOH (9:1). The left panel shows the sample extract, and the right panel shows the solvent control. The red circle highlights the zone of inhibition indicating antifungal activity. (**B**) Schematic workflow illustrating the extraction and purification steps of compound **1** from strain BL11. (**C**) HPLC profile showing the peak corresponding to compound **1**.

Considering the strong inhibitory effect of strain BL11 on *A. fumigatus*, we carried out the fermentation process of strain BL11. The aim was to extract the secondary metabolites from the resulting fermentation broth. To determine the fraction with antifungal activity, bioautography assays were conducted. In these assays, 2 mg of two distinct extracts were utilized. The findings of the assays showed that only the fraction having an Rf value of 0.61 presented inhibition zones (Fig. 2A). After that, the crude extract underwent purification procedures. First, silica gel column chromatography was applied, followed by high-performance liquid chromatography (HPLC). Through these purification steps, compound **1** was successfully isolated (Fig. 2B and C).

Compound **1** was obtained in the form of a white powder. To determine its chemical structure, high-resolution electrospray ionization mass spectrometry (HR-ESI-MS) was employed. Based on the [M+Na]^+^ ion signal at *m/z* 587.3559, the molecular formula of compound **1** was deduced to be C_32_H_52_O_8_. This formula implies that the compound has 7 degrees of unsaturation (Fig. S2). Further structural analysis was carried out using the ^13^C NMR spectrum in combination with the Distortionless Enhancement by Polarization Transfer analysis. The results of this analysis revealed the presence of 32 carbon resonances. Specifically, these resonances consisted of 4 methyl groups, 7 methylene groups, 20 methine groups, and 1 quaternary carbon (Fig. S3). Moreover, the existence of a long conjugated double bond within the structure of compound **1** was verified. This was evidenced by the observation of several overlapping methine signals (δ_H_ 6.07–6.27) in the low magnetic field region of the NMR spectrum (Table S1).

**Fig 3:**
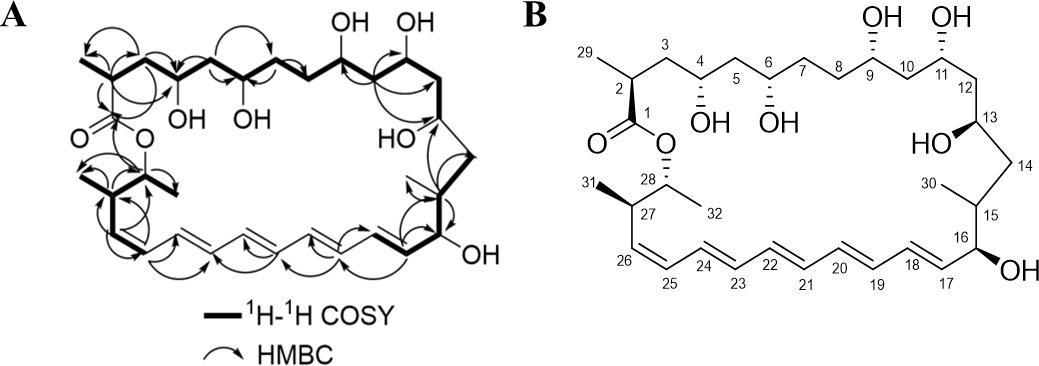
Structure identification of compound **1**. (**A**) Key HMBC and COSY correlations of compound **1**. (**B**) The spatial structure of compound **1**.

The ^1^H (Fig. S4) and ^13^C NMR (Fig. S3) spectra of **1** in conjunction with the HSQC (Fig. S5), ^1^H-^1^H COSY (Fig. S6), and HMBC (Fig. S7) spectra suggested the presence of six hydroxy methines (δ_C_ 67.67, 70.22, 71.90, 72.50, 74.71, and 75.20) and of a conjugated pentaene (H-14 to H-18 and H-25 to H-28). In addition, the ^1^H-^1^H COSY correlations with olefinic protons indicated that one hydroxy methine carbon (δ_C_ 75.20) was assigned to C-16. The COSY spectrum of 1 showed four ^1^H spin systems of H-2/H-3/H-4/H-5/H-6/H-7, H-8/H-9/H-10/H-11/H-12/H-13, H-14/H-15/H-16/H-17/H-18, and H-25/H-26/H-27/H-28. The HMBC experiment (Fig. S7) showed that the methine proton at δ_H_ 2.13 (H-2) correlated with the carbons at δ_C_ 173.65 (C-1), 22.63 (C-3), 71.90 (C-4), 42.12 (C-5), 72.01 (C-6), and 11.84 (C-29); the methylene proton at δ_H_ 1.75 (H-3a) correlated with the carbons at δ_C_ 173.65 (C-1), 54.63 (C-2), 71.90 (C-4), 72.01 (C-6), and 11.84 (C-29); the methylene proton at δ_H_ 1.24 (H-5) correlated with the carbons at δ_C_ 54.63 (C-2), 71.90 (C-4), 72.01 (C-6), and 38.54 (C-7); the methyl proton at δ_H_ 1.05 (H-7) correlated with the carbons at δ_C_ 71.90 (C-4), 42.12 (C-5), 72.01 (C-6), and 23.08 (C-8); methylene proton δ_H_ 1.31 (H-10a) and 1.11 (H-10b) correlated with the carbons at δ_C_ 72.50 (C-9), 70.22 (C-11), 39.18 (C-12), and 67.67 (C-13); the methine proton δ_H_ 1.58 (H-15) correlated with the carbons at δ_C_ 67.67 (C-13), 39.41 (C-14), 75.20 (C-16), and 10.70 (C-30); the methine proton δ_H_ 5.54 (H-17) correlated with the carbons δ_C_ 45.93 (C-15), 75.20 (C-16), and 133.30 (C-19); the methine proton δ_H_ 6.18 (H-19) correlated with the carbons δ_C_ 137.01 (C-17), 131.13 (C-18), 133.61 (C-20), 131.92 (C-21), and 132.40 (C-22); the methine proton δ_H_ 6.27 (H-21) correlated with the carbons δ_C_ 132.40 (C-22) and 131.69 (C-23); the methine proton δ_H_ 6.11 (H-25) correlated with the carbons δ_C_ 133.61 (C-20), 131.69 (C-23), 133.64 (C-24), and 39.93 (C-27); the methine proton δ_H_ 5.91 (H-26) correlated with the carbons δ_C_ 133.64 (C-24), 129.70 (C-25), 39.93 (C-27), and 74.71 (C-28); the methine proton δ_H_ 2.36 (H-27) correlated with the carbons δ_C_ 129.70 (C-25), 137.02 (C-26), 74.71 (C-28), 16.10 (C-31), and 19.51 (C-32); the methine proton δ_H_ 4.63 (H-28) correlated with the carbons δ_C_ 173.65 (C-1), 137.02 (C-26), 39.93 (C-27), 16.10 (C-31), and 19.51 (C-32), meaning that compound **1** forms a cyclic structure here, and compound **1** was determined to be a 28-membered polyene macrolide (Fig. 3A). The NOESY (Fig. S8) experiment showed NOE interactions between H-4-OH, H-6-OH, H-9-OH, H-11-OH, H-13-OH, H-16-OH, and H-9, together with a biogenetic perspective (14, 15), and the absolute configurations of compound **1** were proposed to be 2S, 4R, 6S, 9S, 11S, 13R, 16R, 27R, and 28R (Fig. 3).

### Antifungal activity of compound 1 against pathogenic fungi

Compound **1**, isolated from strain BL11, demonstrated potent antifungal activity against a panel of clinically relevant fungal pathogens, including *A. fumigatus*, *A. flavus*, *Candida albicans*, and *Cryptococcus neoformans*. To evaluate its efficacy, fungal growth was monitored over 24 h following treatment with varying concentrations of compound **1**. The minimum inhibitory concentration (MIC) varied among species and strains. For the reference strain *A. fumigatus* Ku80 and the azole-resistant clinical isolate C16 (resistant to isavuconazole [ISA] and voriconazole [VOR]), the MIC was 16 μg/mL. In contrast, the MIC for strain C96, resistant to itraconazole (ITR) and ISA, was 8 μg/mL.

**Fig 4:**
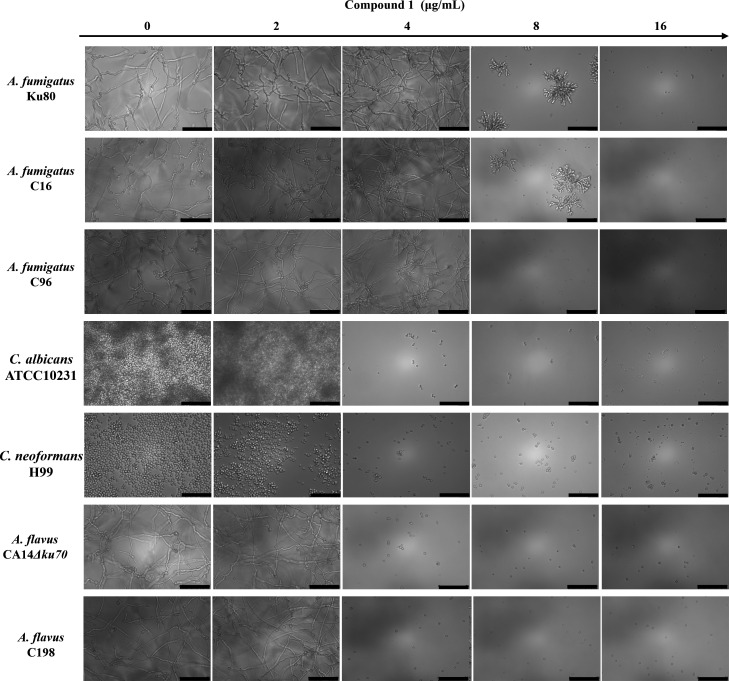
Antifungal activity of compound **1** against diverse fungal species. MIC assays were conducted in RPMI 1640 medium using twofold serial dilutions of compound **1**. Tested fungal strains included *A. fumigatus* C16 (resistant to ISA and VOR), *A. fumigatus* C96 (resistant to ISA and ITR), and *A. flavus* C198 (resistant to ISA, AMB, and VOR). Scale bar = 75 μm.

In addition, compound **1** exhibited strong activity against the multidrug-resistant *A. flavus* strains CA14Δ*ku70* and C198 (resistant to amphotericin B [AMB], ISA, and VOR), *C. albicans* ATCC10231, and *C. neoformans* H99, with an MIC of 4 μg/mL in each case (Fig. 4). These findings underscore the potential of compound **1** as a broad-spectrum antifungal agent, including efficacy against drug-resistant fungal strains.

### Dose-dependent inhibition of *A. fumigatus* growth by compound 1

**Fig 5:**
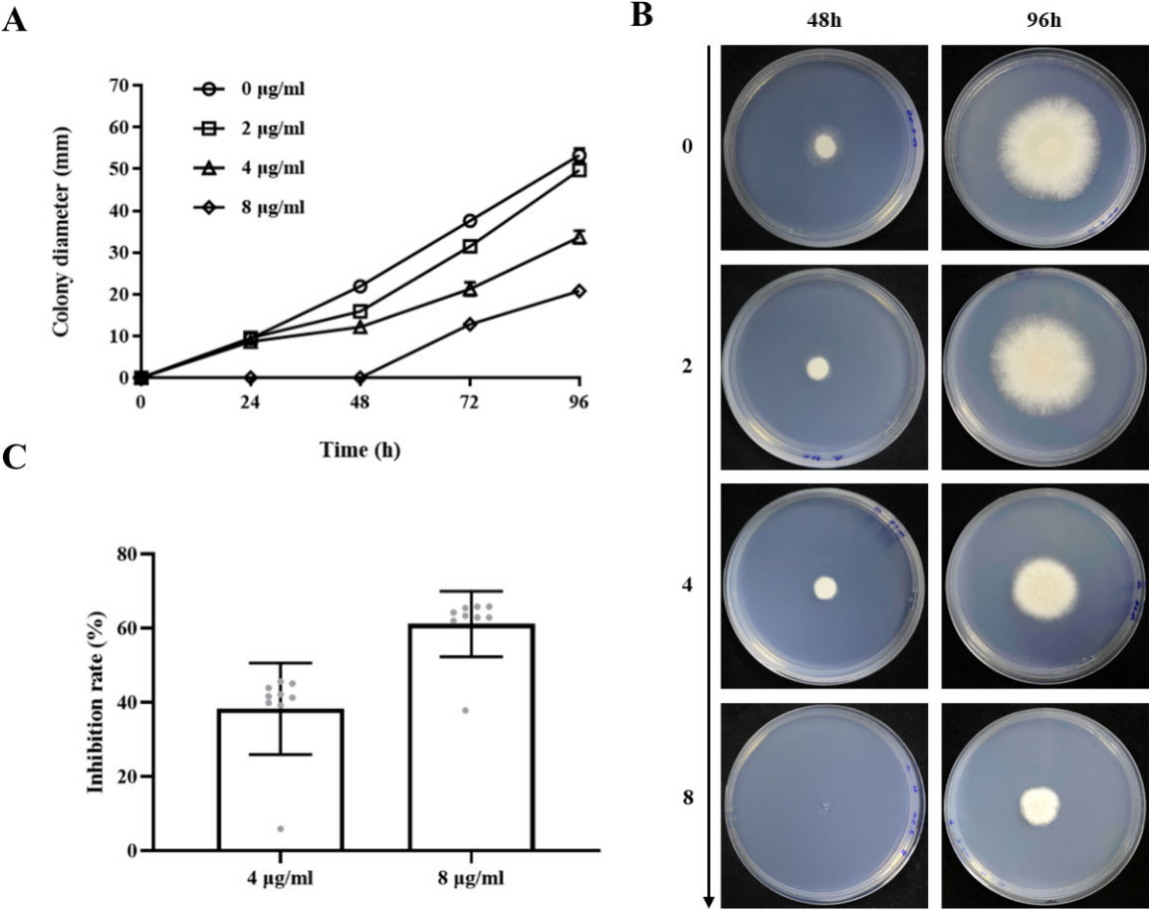
Effect of compound **1** on the growth of *A. fumigatus*. (**A**) Radial colony diameters were measured at 24-h intervals to assess growth kinetics. (**B**) Colony morphology of *A. fumigatus* after treatment with 0, 2, 4, or 8 μg/mL of compound **1** for 48 and 96 h. (**C**) Inhibition rates of *A. fumigatus* growth following 96 h of exposure to compound **1** at different concentrations.

Building on its broad-spectrum antifungal efficacy, the inhibitory effect of compound **1** against *A. fumigatus* was further investigated in detail. Drug susceptibility was assessed using agar plates supplemented with various concentrations of compound **1**. Treatment with 2 and 4 μg/mL resulted in moderate growth suppression compared to the untreated control (Fig. 5A). At 8 μg/mL, no visible colonies were observed during the first 48 h. Colonies that eventually emerged showed markedly reduced growth relative to the control (Fig. 5A and B). A clear dose-dependent inhibition of radial growth was observed, with inhibition rates reaching 38% at 4 μg/mL and 61% at 8 μg/mL after 96 h of incubation (Fig. 5B and C). These results confirm that compound **1** inhibits *A. fumigatus* growth in a concentration-dependent manner.

### Transcriptomic profiling reveals global gene expression changes in *A. fumigatus* induced by compound **1**

**Fig 6:**
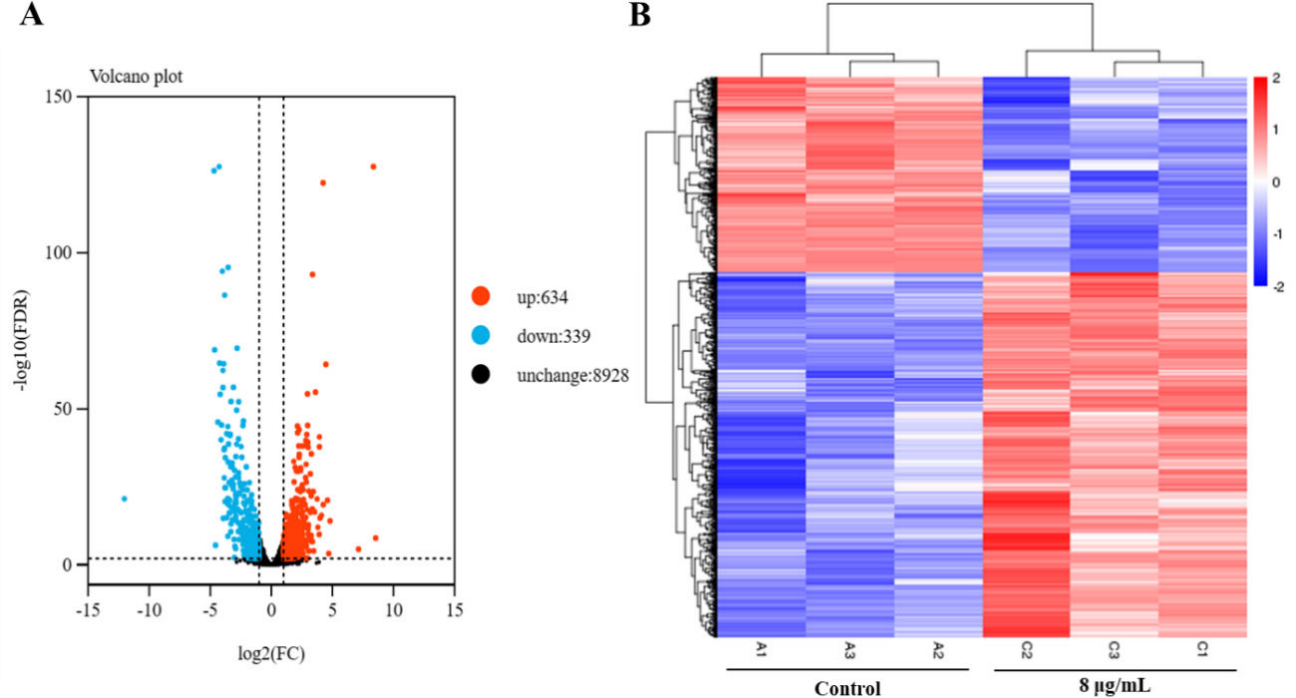
Analysis of DEGs in *A. fumigatus* mycelia following compound **1** treatment. (**A**) Volcano plot showing the distribution of DEGs in *A. fumigatus* treated with compound **1**. Downregulated genes are indicated in blue, upregulated genes in red, and non-significant genes in black. (**B**) Heat map depicting hierarchical clustering of DEGs. Red indicates upregulation, while blue indicates downregulation in response to compound **1** treatment.

To elucidate the molecular mechanisms underlying the antifungal activity of compound **1**, we conducted the transcriptomic analysis of *A. fumigatus* following exposure to the compound. Treatment with compound **1** resulted in significant transcriptional reprogramming, with a total of 973 differentially expressed genes (DEGs) identified—comprising 634 upregulated and 339 downregulated genes—when compared to the untreated control. The distribution and clustering of these DEGs were illustrated using a volcano plot and a hierarchical heat map (Fig. 6A and B).

**Fig 7:**
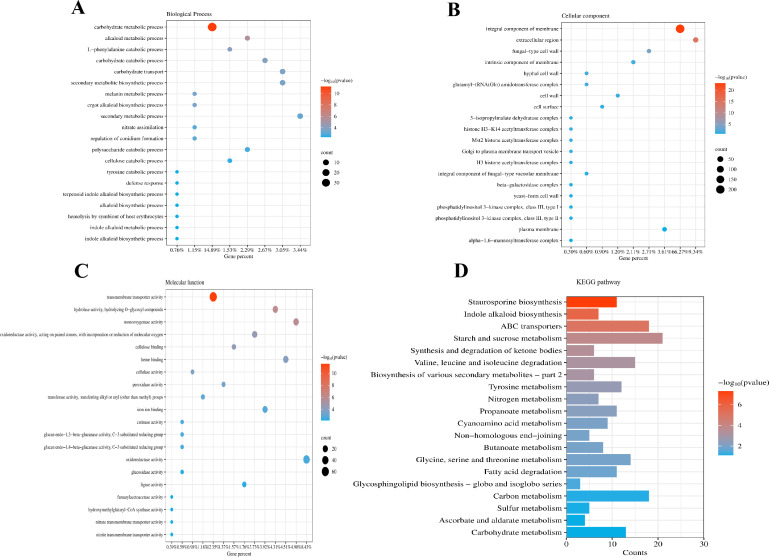
GO enrichment analysis and KEGG pathway analysis of DEGs in *A. fumigatus* treated with compound **1**. (**A**) Top 20 biological processes terms in GO categories. (**B**) Top 20 cellular component terms in GO categories. (**C**) Top 20 molecular function terms in GO categories. (**D**) Top 20 KEGG pathways.

Gene Ontology (GO) enrichment analysis revealed that the identified DEGs were associated with various biological processes and molecular functions, including carbohydrate metabolism, integral membrane components, transmembrane transporter activity, plasma membrane organization, oxidoreductase activity, and monooxygenase activity (Fig. 7A through C). Additionally, Kyoto Encyclopedia of Genes and Genomes (KEGG) enrichment analysis indicated that most DEGs were involved in ABC transporters pathways, starch and sucrose metabolism, and carbon metabolism (Fig. 7D). These results suggest that compound **1** disrupts key aspects of *A. fumigatus* cellular homeostasis, particularly affecting membrane function, metabolic processes, and transport systems.

To further elucidate the inhibitory effects of compound **1** on *A. fumigatus* growth, representative DEGs were categorized into three groups: oxidative stress, transmembrane transport, and cell wall/membrane (Table S2). Notably, significant changes were observed in genes associated with cell wall and membrane biogenesis, including *ags2*, *ags3*, *Scw4*, *eng4*, *eng5*, *chi5*, *Afu5g00670*, *Afu5g02130*, and *Afu8g01130*. In addition, genes encoding antioxidant-related enzymes, such as catalase (CAT, *Afu2g18030*), peroxidase (POD, *Afu5g02300* and *Afu5g15070*), glutathione S-transferase (GST, *Afu4g01440*), and oxidoreductases (*Afu1g15610*), were significantly affected (Table S3). Furthermore, compound **1** suppressed the expression of key genes involved in asexual reproduction in filamentous fungi, including *brlA*, *wetA*, and *abaA* (Table S2).

### Compound 1 disrupts the cell morphology and compromises cell wall integrity

**Fig 8:**
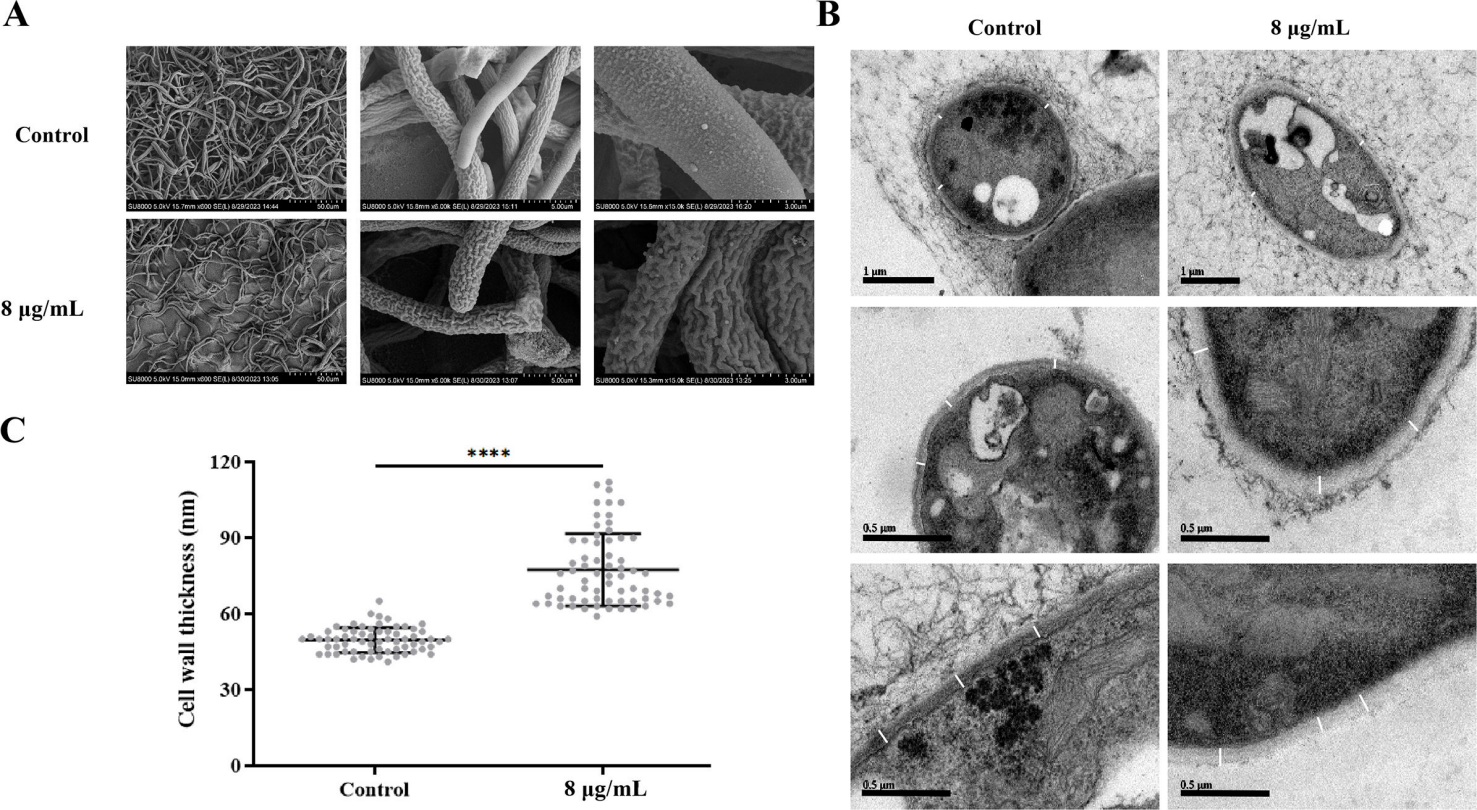
Compound **1** affects the morphology, cell wall, and cell membrane of *A. fumigatus*. (**A and B**) Representative SEM and transmission electron microscopy (TEM) images of *A. fumigatus* hyphae after treatment with compound **1**, showing disrupted surface morphology and ultrastructural damage to the cell wall and membrane. (**C**) Quantification of cell wall thickness. At least 15 measurements were taken per cell, with a total of ≥60 cells analyzed per condition. Statistical significance was assessed by multiple *t*-tests (****P < 0.0001).

To assess the impact of compound **1** on the overall morphology of *A. fumigatus*, we performed scanning electron microscopy (SEM). In the SEM micrographs, untreated hyphae appeared normal, with a turgid and smooth surface. In contrast, hyphae treated with compound **1** exhibited a severely rough, wrinkled, and flaccid outer surface (Fig. 8A).

Given the transcriptional changes observed in genes involved in cell wall and membrane biogenesis, we conducted further analyses. These cellular components are essential for fungal growth and survival, so we examined their structural integrity in more detail. TEM was used to examine ultrastructural changes in *A. fumigatus* cells. Untreated cells displayed intact cell walls and membranes with well-defined boundaries. However, compound **1** treatment led to severe structural abnormalities, including disorganized cell wall and membrane architecture, fuzzy boundaries, and a lack of distinct demarcations (Fig. 8B). Additionally, quantitative analysis using ImageJ revealed a significant increase in cell wall thickness in the treated group compared to the control group (Fig. 8C).

### Compound 1 disrupts cell membrane integrity

**Fig 9:**
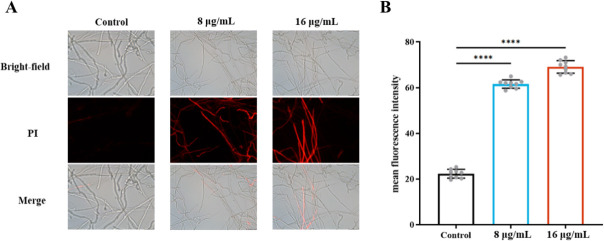
Cell membrane damage in *A. fumigatus* following compound **1** treatment. (**A**) *A. fumigatus* cells were treated with 8 μg/mL or 16 μg/mL compound **1** for 2 h, followed by PI staining. Fluorescence images were acquired using a Leica DM6B microscope. (**B**) Mean fluorescence intensity was quantified using ImageJ. Statistical significance was assessed by multiple *t*-tests (****P < 0.0001).

To validate the transcriptional evidence suggesting downregulation of genes involved in cell membrane biogenesis, we assessed membrane integrity using propidium iodide (PI) staining. PI is a fluorescent dye that penetrates cells with compromised membranes and binds to nucleic acids. In *A. fumigatus* treated with compound **1**, strong PI fluorescence was observed, in contrast to the weak signal in untreated controls, indicating increased membrane permeability (Fig. 9A). Moreover, the fluorescence intensity increased in a dose-dependent manner with higher concentrations of compound **1**, suggesting progressive disruption of membrane integrity (Fig. 9B).

### Induction of reactive oxygen species (ROS) levels and antioxidant enzyme activities

**Fig 10:**
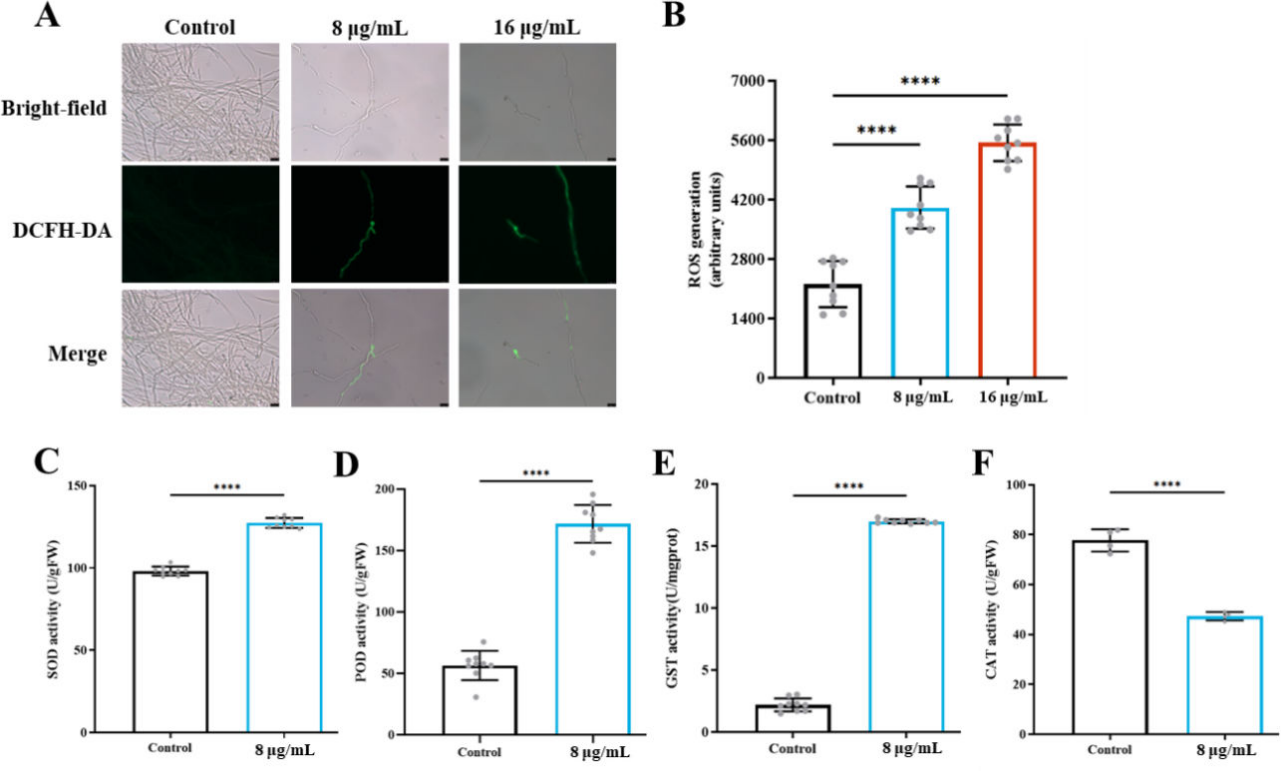
Effect of compound **1** on ROS accumulation and antioxidant enzyme activities in *A. fumigatus*. (**A**) ROS levels were assessed using DCFH-DA staining after treatment with 8 μg/mL or 16 μg/mL compound **1**. (**B**) Quantification of fluorescence intensity using a microplate reader at 560 nm to evaluate ROS accumulation. (**C–F**) Activities of antioxidant enzymes following compound **1** treatment: (**C**) superoxide dismutase (SOD); (**D**) POD; (**E**) GST; and (**F**) CAT. Statistical significance was assessed by multiple *t*-tests (****P < 0.0001).

Eukaryotic microorganisms typically activate antioxidant defense systems when exposed to oxidative stress. To determine whether compound **1** induces oxidative stress in *A. fumigatus*, we first monitored intracellular ROS levels using 2′,7′-dichlorodihydrofluorescein di-acetate (DCFH-DA) staining. Treatment with 8 μg/mL or 16 μg/mL compound 1 resulted in markedly increased green fluorescence, reflecting the oxidation of the non-fluorescent probe 2′,7′-dichlorodihydrofluorescein (DCFH) to its fluorescent product, 2′,7′-dichlorofluorescein (DCF), by intracellular ROS (Fig. 10A and B). This indicates that compound **1** elevates ROS levels in *A. fumigatus* cells.

To further evaluate oxidative stress responses, we quantified the activities of key antioxidant enzymes, including SOD, POD, GST, and CAT (16). Consistent with ROS accumulation, compound 1 significantly enhanced SOD (1.30-fold), POD (3.04-fold), and GST (7.73-fold) activities compared with untreated controls (Fig. 10C through E). In contrast, CAT activity decreased to 0.39-fold of control levels (Fig. 10F). Together, these findings demonstrate that compound 1 promotes ROS accumulation and disrupts the antioxidant defense balance in *A. fumigatus*, which likely contributes to its antifungal activity.

## DISCUSSION

Fungal infections pose a significant and growing global health challenge, exacerbated by the limited arsenal of antifungal drugs and rising resistance (17, 18). Natural products remain a valuable source of new antifungal agents, underscoring the need to identify novel bioactive scaffolds and elucidate their mechanisms of action (19). In this study, we aimed to identify novel antifungal candidates. From forest soil samples, we isolated a *Kitasatospora melanogena* strain and subsequently purified a bioactive metabolite, designated compound **1**. We characterized both its antifungal activity and its mechanism of action. *Kitasatospora* species are well-known producers of bioactive compounds, including antifungals and antibacterials, with over 50 such compounds reported to date (20). Large-scale fermentation of strain BL11 followed by fractionation and purification via chromatography led to the isolation of the most bioactive fraction, ultimately yielding compound **1**. This compound exhibited broad-spectrum antifungal activity, significantly inhibiting the growth of *A. fumigatus*, *A. flavus*, *C. albicans*, and *C. neoformans* (Fig. 4), all of which are major fungal pathogens responsible for aspergillosis, candidiasis, and cryptococcal meningitis. Given the increasing prevalence of fungal infections and antifungal resistance, these findings highlight the therapeutic potential of compound **1** as a broad-spectrum antifungal.

Understanding the mechanisms of natural antifungal agents is essential for their effective application in combating fungal diseases. The fungal cell wall serves as a crucial structural component, maintaining cell morphology and acting as a protective barrier (21). TEM analysis (Fig. 8B) revealed that treatment with compound **1** at 8 μg/mL led to significant thickening of the *A. fumigatus* cell wall compared to the control group. The treated cells exhibited a loosened and heterogeneously thickened cell wall, a commonly observed adaptive response of pathogenic fungi under antifungal stress (22, 23). Quantification of cell wall thickness (Fig. 8C) further confirmed this alteration, showing a statistically significant increase in the treated group (*P* < 0.0001). This conclusion is supported by at least 15 measurements per cell and a minimum of 60 cells analyzed per condition. The observed thickening likely reflects compensatory remodeling, involving abnormal deposition of structural polysaccharides such as glucans and chitin. Transcriptomic analysis further corroborates these morphological changes, revealing significant downregulation of *ags2* and *ags3*, which encode α-glucan synthases essential for cell wall integrity and virulence (24). Because α-glucan is a major structural component required for cell morphology, osmotic stability, and immune evasion (25), reduced glucan biosynthesis is expected to compromise normal wall architecture. Interestingly, despite the transcriptional suppression of glucan synthase genes, TEM analysis revealed a thickened cell wall. This suggests the activation of a compensatory stress response, in which fungal cells increase chitin deposition to reinforce the cell wall when glucan networks are impaired (26). Similar stress-induced remodeling has been reported in *A. fumigatus* and other fungal pathogens upon exposure to echinocandin antifungals, wherein β-glucan inhibition triggers enhanced chitin deposition (22). This abnormal accumulation may explain the irregularly thickened and structurally loosened appearance of the cell wall in compound **1**-treated cells. Taken together, these findings suggest that compound **1** exerts its antifungal effects by inhibiting α-glucan biosynthesis, thereby disrupting cell wall integrity and inducing a compensatory remodeling response. This dual mechanism likely impairs *A. fumigatus* growth and adaptation, providing novel insights into its antifungal activity. Further studies, including cell wall composition analysis (e.g., quantification of glucans and chitin) and gene knockout experiments, are warranted to confirm the molecular targets of compound **1** and assess its potential for antifungal therapy.

The fungal cell membrane is essential for nutrient exchange, stress adaptation, signal transduction, and immune responses. Disruption of membrane integrity can severely compromise conidial viability and overall fungal survival (27). In this study, transcriptomic analysis revealed pronounced downregulation of genes involved in membrane structure and function, and microscopy confirmed extensive membrane damage. PI staining further validated this phenotype: compound 1-treated cells displayed strong, dose-dependent fluorescence, indicating increased membrane permeability. These results suggest that compound 1 directly compromises membrane integrity, thereby facilitating compound entry and enhancing antifungal activity.

Integral membrane proteins (IMPs) are crucial for vesicle trafficking, protein transport, and cellular communication (28, 29). Consistent with membrane disruption, genes encoding IMPs (*Afu8g00670*, *Afu6g04280*, and *Afu2g17760*) were significantly downregulated following compound 1 exposure, similar to effects reported for membrane-targeting antifungals (30). In addition, multidrug resistance (MDR) transporters—including ABC and major facilitator superfamily (MFS) transporters—mediate drug efflux and contribute to antifungal resistance (31, 32). Compound **1** treatment reduced the expression of multiple ABC (*Afu3g03430, Afu4g01050, Afu3g03670,* and *atrF*) and MFS (*Afu5g10430, Afu4g06050, Afu4g00570, Afu3g02060,* and *Afu4g11780*) transporter genes. This mirrors reports that certain flavonoids inhibit microbial efflux pumps, suggesting that compound 1 may increase its intracellular retention by suppressing MDR transporters (30).

ROS accumulation is another key component of antifungal action (33), and many clinically used antifungals—such as AMB, ITR, and terbinafine—exert toxicity through ROS induction (34–36). Compound **1** similarly elevated intracellular ROS levels, as shown by enhanced DCF fluorescence (Fig. 9A and B), consistent with ROS-mediated antifungal mechanisms reported for natural products such as chitosan (37). In response, *A. fumigatus* activated its antioxidant defense pathways: SOD, POD, and GST activities increased significantly (38). Correspondingly, peroxidase (Afu5g02300) and GST (Afu4g01440) genes were upregulated, resulting in ~3-fold and ~8-fold increases in enzyme activity, respectively. Genes encoding thioredoxin (Afu5g15070) were also induced (Table S3).

Despite its promising antifungal activity, compound **1** presents notable limitations. Structurally, it shares significant similarity with AMB (39), particularly a polyene region (C16–C32) containing five conjugated double bonds, which are prone to photooxidation and spontaneous degradation—factors that may compromise its stability. Similar to AMB, stabilizing strategies such as antioxidant co-formulation or chemical modifications (40) to reduce oxidation susceptibility may help preserve the structural integrity and prolong the pharmacological activity of compound **1**.

In addition, glycosylation is a well-established strategy for enhancing antifungal potency and selectivity. For example, mandimycin contains three glycosyl units, including atractylose A, which is essential for its full antifungal activity (41). Loss of this moiety in mandimycin B leads to a marked reduction in efficacy and target binding affinity. Since no glycosylation has been identified in compound **1**, introducing specific glycosyl groups may improve its bioactivity, membrane interaction, and target specificity. Such structural modifications could broaden the therapeutic potential of compound **1** and support its development as a novel antifungal agent.

**Fig 11:**
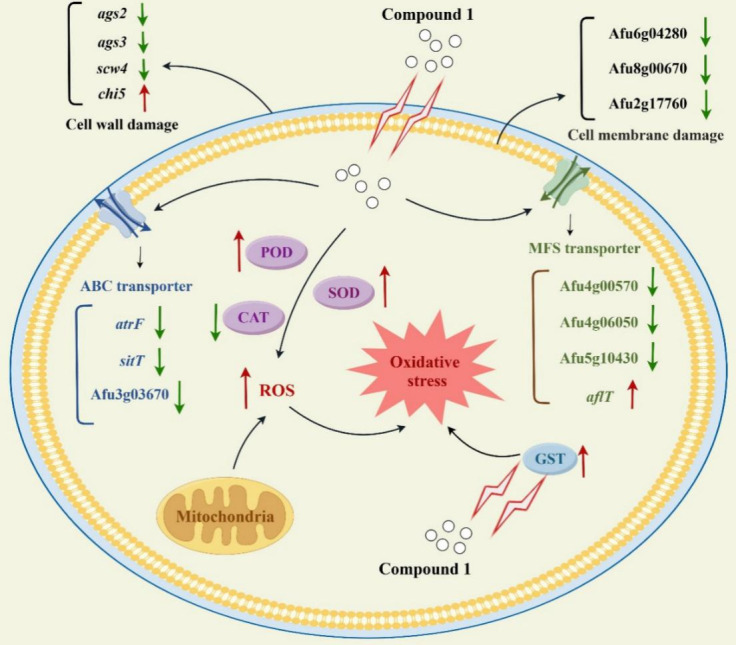
Schematic model of the antifungal mechanism of compound **1** against *A. fumigatus*. The illustration was created using Figdraw (https://www.figdraw.com/#/).

In summary, our study identified compound **1**, a novel antifungal agent from *K. melanogena* strain BL11, with broad-spectrum activity against major fungal pathogens. Mechanistic studies showed that it disrupts fungal cell wall integrity by inhibiting α-glucan biosynthesis and induces compensatory chitin deposition. It also compromises membrane integrity and suppresses MDR transporters, enhancing intracellular accumulation (Fig. 11). Additionally, compound **1** triggers ROS accumulation and antioxidant responses, contributing to its antifungal effects. However, its structural instability may limit clinical application. Future modifications, such as glycosylation or formulation strategies, could improve its stability and bioavailability. These findings lay the groundwork for further development of compound **1** as a promising antifungal candidate.

## MATERIALS AND METHODS

### Strains and culture conditions

The *A. fumigatus* Ku80, *A. flavus* CA14Δ*ku70, C. albicans* ATCC10231, and *C. neoformans* H99 stored in our laboratory were used in this study. The clinical isolates of *A. fumigatus* (C16 and C96) and *A. flavus* C198 were isolated from hospitalized patients with fungal infections from the Jiangxi Provincial People’s Hospital. *A. fumigatus* and *A. flavus* were inoculated on PDA medium and cultured at 37°C for 2 days to collect spores. *C. albicans* and *C. neoformans* were inoculated in YPD liquid medium and cultured at 37°C for 24 h to collect cells.

### Collection of soil samples and isolation of strains

The strains were isolated from soil samples collected in Banlao, Yunnan, China, utilizing a concentration gradient dilution method (33) Briefly, 5 g of soil samples were dried at 40°C for about 6 h and then mixed with 10 mL of sterile water in 15 mL sterilized centrifuge tubes. The resultant suspension was vigorously vortexed at 28°C and 150 rpm for 30 min. The soil suspension was then subjected to gradient dilution to obtain dilutions ranging from 10^−1^ to 10^−7^. Subsequently, 100 µL of the 10^−5^, 10^−6^, and 10^−7^ dilutions were spread onto TWYA, G1, and ISP2 isolation media and incubated at 28°C for 7 days. Single colonies were selected and transferred to G1 agar plates for further isolation and purification. The isolates underwent screening using the plate confrontation method. *Streptomyces* strains were inoculated on both sides of G1, YPD, and YAG plates. After incubation at 28°C for 7 days, the antagonistic interaction between *Streptomyces* and *A. fumigatus* was observed by adding 2 μL of 10^7^ spores of *A. fumigatus* to the center of the plates.

### Morphological and genotypic identification of strain BL11

The strain was cultured on G1 agar medium at 25°C for 7 days, and its growth status and hyphal morphology were observed under a microscope with a 40× objective lens. For genotypic identification of strain BL11, 16S rRNA sequencing was employed. Genomic DNA was extracted using the TIANGEN DNA extraction kit (TIANGEN Biotech Co., Ltd., Beijing, China). The 16S rRNA gene sequence was amplified using the universal primers 27F (5′-AGAGTTTGATCCTGGCTCAG-3′) and 1495R (5′-CTACGGCTACCTTGTTACGA-3′). Amplification was carried out in a 50 μL reaction volume, which included 25 μL of 2× Taq PCR Mix, 1 μL each of forward and reverse primers, 1 μL of the DNA template, and 22 μL of ddH_2_O. The PCR conditions were as follows: initial denaturation at 96°C for 3 min, followed by 30 cycles of denaturation at 94°C for 10 s, annealing at 58°C for 15 s, and extension at 72°C for 1 min. The PCR products were verified by agarose gel electrophoresis, purified, and sequenced at QIAGEN Biotech (Guangzhou). The sequences were analyzed for similarity to other known sequences in the GenBank database using BLAST, and a phylogenetic tree was constructed using MEGA-X software.

### Fermentation culture of the BL11 strain and purification of its crude extract

Strain BL11 was grown in YAG medium (2 L) at 28°C for 14 days. After the incubation period, the fermentation products of strain BL11 were extracted three times with ethyl acetate (EtOAc) to obtain the crude extract.

### TLC and bioautography-based detection of bioactive fraction

Bioautography was carried out using TLC to identify which fraction of the extract contained compounds exhibiting antifungal activity. The extract, dissolved in methanol, was applied to a TLC plate and separated using the methanol: chloroform (9:1) eluent system. The separation of the compounds on the TLC was visualized under UV light at 254 nm. Mobile phase methanol: chloroform (9:1) was also placed on the TLC plate as solvent control. The bioautography procedure followed the previously described method (42). The TLC plate with separated compounds was overlaid with an agar medium inoculated with *A. fumigatus* spores to detect the antifungal activity. After incubation, zones of inhibition on the agar indicated the presence of antifungal compounds. To further isolate the active compounds, the crude extract in EtOAc was subjected to silica gel column chromatography (22×3 cm). Five fractions (Fr.1–Fr.5) were prepared using a gradient solvent system of dichloromethane-methanol (20:1, 10:1, 9:1, 8:2). Following activity-guided fractionation, fraction 4 (Fr.4) exhibited antifungal activity. Fr. 4 was then purified using semi-preparative HPLC with 78% methanol as the mobile phase over a 20-min run to isolate compound **1**. This approach ensured that the active antifungal compound was accurately identified and isolated for further analysis.

### Structure elucidation of compound 1

The NMR spectroscopy was performed to determine the structure of compound **1** by using Agilent NMR system 800 MHz NMR spectrometer (Agilent Technologies Inc., Colorado Springs, CO, USA). To confirm the molecular weight and structural details, electrospray ionization mass spectrometry and HR-ESI-MS were performed using a Waters Xevo G2-S QTOF MS spectrometer (Waters, Milford, MA, USA).

Compound **1**: a white powder; [α]^25^_D_ = −60.0 (c = 0.1, MeOH); UV (MeOH) λ_max_ (log ε): 349 (1.493), 331 (1.467), 316.5 (1.248) (Fig. S1). ^1^H and ^13^C data (Table S1); HR-ESI-MS: m/z 587.3559 ([M+Na]^+^, calc. 587.3560) (Fig. S2).

### Purity verification, storage, and usage of compound 1

Compound 1 was purified to analytical purity through repeated chromatographic steps and confirmed by NMR, HR-ESI-MS, UV spectroscopy, and optical rotation measurements. All data demonstrated a single, well-defined structure with no detectable impurities. To minimize degradation risk, all samples were handled under low-light conditions, stored at −20°C, and freshly prepared in appropriate solvents immediately prior to use.

### *In vitro* determination of antifungal activity

The antifungal activity of compound **1** was assessed using the MIC test, followed by a checkerboard assay with the microdilution method as previously reported (43). Each well of a 96-well microtiter plate was filled with 100 μL of fresh spore suspension (2 × 10^5^ spores/mL). Subsequently, 96 μL of RPMI 1640 liquid medium and 4 μL of compound **1** stock solution were added to achieve final concentrations ranging from 0 to 64 μg/mL. Following incubation at 37°C for 24 h, the MIC of compound **1**, defined as the lowest concentration inhibiting spore germination within 24 h, was determined. DMSO (Sigma-Aldrich) was used as the solvent for all compounds.

### Growth assay

To assess the effect of compound **1** on the growth of *A. fumigatus*, 10 μL of the spore suspension at a concentration of 10^8^ spores/mL was inoculated onto MM agar plates containing compound concentrations of 0, 2, 4, and 8 μg/mL. The plates were then incubated at 37°C, with colony diameters recorded every 12 h over a duration of 5 days. Colony morphology was photographed on days 2 and 4 post-inoculation.

### Microscopy analysis

The mycelial morphology after treatment with compound **1** was determined by SEM according to a previously reported method (44). The prepared fungal mycelia were fixed with 3% glutaraldehyde and incubated in darkness at 4°C for more than 24 h. The fixative was then discarded, and the mycelia were washed with 3× phosphate-buffered saline (PBS), followed by lyophilization. The samples were then coated with gold powder to make them conducive for SEM analysis.

To examine the morphological characteristics of the mycelial cell wall, TEM was employed as described previously (42). In brief, a 10^7^ conidial suspension was inoculated onto MM agar plates containing concentrations of compound **1** at 0 μg/mL and 8 μg/mL. The freshly harvested mycelia were fixed in phosphate-buffered glutaraldehyde, followed by treatment with osmium tetroxide. After triple washing with ddH_2_O, the samples underwent stepwise dehydration using acetone. Subsequently, the dehydrated samples were embedded in Epon 812 resin for sectioning. Ultrathin sections were then stained with uranium acetate and examined using a JEOL JEM-1400 transmission electron microscope operating at 120 kV.

### Comparative transcriptomic analysis

To assess transcriptomic changes in *A. fumigatus* upon compound 1 treatment, conidia of strain ku80 were inoculated onto MM agar plates with 0 (control) or 8 μg/mL of compound 1. Mycelia were harvested at 24 and 48 h and immediately frozen. Three biological replicates were collected per condition. Total RNA was extracted and quality-checked (RIN ≥ 7). Strand-specific cDNA libraries were prepared using the NEBNext Ultra RNA Library Prep Kit (NEB, USA), clustered on a cBot system (TruSeq PE Cluster Kit v4), and sequenced on an Illumina platform (150 bp paired-end reads). Clean reads were aligned to the reference genome using HISAT2, and gene expression was quantified with featureCounts (FPKM). Differential expression was analyzed with DESeq2 and EBSeq (fold change > 2, FDR < 0.01). GO and KEGG enrichment analyses used hypergeometric tests with Benjamini–Hochberg FDR correction. Selected DEGs can be validated by qRT-PCR in future studies.

Gene functions were annotated using several databases including the following: Nr (NCBI non-redundant protein sequences), Nt (NCBI non-redundant nucleotide sequences), Pfam (protein family), KOG/COG (Clusters of Orthologous Groups of proteins), Swiss-Prot (a manually annotated and reviewed protein sequence database), KO (KEGG Ortholog database), and GO (Gene Ontology). Additionally, statistical enrichment of differentially expressed genes in KEGG and GO pathways was tested using the online tool available at https://www.bioinformatics.com.cn/.

### Cell membrane integrity determination

To assess plasma membrane integrity, PI staining was employed, following the method described by Xu et al. (45). Fungal samples were treated and collected, as previously outlined, and then stained with 10 μg/mL PI for 20 min in the dark at 37°C. After staining, samples were centrifuged, re-suspended in PBS, and examined using laser confocal microscopy.

### Assessment of ROS levels

To quantify ROS levels in *A. fumigatus* mycelium, a redox-sensitive fluorescent probe DCFH-DA was used with a ROS detection kit (Beyotime Biotechnology, Shanghai, China). Following the kit instructions, mycelia were incubated with DCFH-DA at 37°C for 20 min. The staining was observed using a Leica DM6B microscope to assess ROS accumulation in compound **1**-treated *A. fumigatus* mycelia.

### Antioxidant enzyme activity assays

Fungal samples were treated and collected as described above, and antioxidant activities were determined. The activity of SOD was measured by monitoring the inhibition of NBT photochemical reduction, following the method described by Giannopolitis and Ries (46), with slight modifications. Briefly, the samples were mixed with 5 mL of 50 mM PBS (pH = 7) and centrifuged at 4°C and 6,000 rpm for 10 min to obtain the crude enzyme extract. Subsequently, 50 μL of the crude enzyme extract was added to 2 mL centrifuge tubes, followed by the addition of 1.55 mL of 50 mM PBS (pH 7.0), 0.1 mL of 1 mg/mL EDTA-Na_2_ solution, 0.1 mL of 20 mg/mL histidine, 0.1 mL of 0.1 mg/mL riboflavin, and 0.1 mL of 1 mg/mL NBT. Light and dark control groups were established, and the mixture was incubated at 28°C for 20 min. The absorbance at 560 nm was then measured.

CAT activity was assessed by monitoring the degradation of H_2_O_2_ at 240 nm as reported previously (47). Using the crude enzyme extract, 0.2 mL of the extract was mixed with 1.5 mL of PBS, 1 mL of dd H_2_O, and 0.3 mL of 0.3% H_2_O_2_. One unit of CAT enzyme activity was defined as the amount required to decompose 1 mmol of H_2_O_2_ per minute at 25°C.

POD and GST activities were determined using commercially available kits from Grace Biotechnology (Suzhou, China) and Jiancheng (Nanjing, China).

All the treatments contained three biological replicates.

### Statistical analysis

Data are shown as mean ± SD. Two-group comparisons were evaluated by two-tailed unpaired Student’s *t*-tests after checking normality (Shapiro–Wilk) and variance homogeneity (Levene); no multiple-comparison correction was needed. RNA-Seq GO/KEGG enrichment used hypergeometric tests with Benjamini–Hochberg FDR correction. A *p* value of <0.05 was considered statistically significant.

## Data Availability

Raw RNA-Seq data (FASTQ files) generated in this study have been deposited in the NCBI Gene Expression Omnibus (GEO) under accession number PRJNA1330178. These data are publicly accessible and can be used to reproduce the analyses presented in this article.

## SUPPLEMENTAL MATERIAL

Supplemental figures and tables spectrum.03143-25-s0001.docx Figures S1 to S8 and Tables S1 to S4.

ASM does not own the copyrights to Supplemental Material that may be linked to, or accessed through, an article. The authors have granted ASM a non-exclusive, world-wide license to publish the Supplemental Material files. Please contact the corresponding author directly for reuse.
