# Supplementary material for: Identification and characterization of a novel broad-spectrum antifungal compound targeting the Aspergillus fumigatus cell wall and cell membrane and inducing oxidative stress
Source: Microbiol Spectr. 2026 Jan 28;14(3):e03143-25. doi: 10.1128/spectrum.03143-25 (PMC12955498; doi:10.1128/spectrum.03143-25)
Supplement: Supplemental figures and tables — Figures S1 to S8 and Tables S1 to S4. [file spectrum.03143-25-s0001.docx]

**Table S1.** The ^1^H-NMR ( ^1^H 800 MHz, δ in ppm, mult. J in Hz), ^13^C-NMR (200 MHz, δ in ppm), HBMC, COSY and NOESY data of compound 1 in DMSO.

| **Position** | **^1^H** | **^13^C** | **HMBC** | **COSY** | **NOESY** |
| --- | --- | --- | --- | --- | --- |
| 1 | - | 173.65, s | - | - | - |
| 2 | 2.13 (1H, td, 10.16, 3.68) | 54.63, d | C-1, C-3, C-4, C-5, C-6, C-29 | H-6, H-3a, H-3b | - |
| 3 | 1.75 (1H, m) | 22.63, t | C-1, C-2, C-4, C-6, C-29 | H-2, H-3b, H-29 | - |
|  | 1.46 (1H, m) |  | C-1, C-2, C-4, C-6, C-29 | H-2, H-29 | - |
| 4 | 3.48 (1H, m) | 71.90, d | C-11 | H-4-OH, H-7, H-12 | - |
| 5 | 1.24 (2H, m) | 42.12, t | C-2, C-4, C-6, C-7 |  | - |
| 6 | 3.64 (1H, t, 9.12) | 72.01, d | - | H-2, H-5, H-6-OH | - |
| 7 | 1.05 (2H, m) | 38.54, t | C-4, C-5, C-6, C-8 | H-4 | - |
| 8 | 1.75 (1H, m) | 23.08, t | C-7 | H-3 | - |
|  | 0.93 (1H, m) |  | C-7 |  | - |
| 9 | 3.44 (1H, m) | 72.50, d | C-11 | H-7, H-9-OH, H-10b, H-12b | - |
| 10 | 1.31 (1H, m) | 43.28, t | C-9, C-11, C-13 | H-11 | - |
|  | 1.11 (1H, m) |  | C-9, C-11, C-12 | H-9 | - |
| 11 | 3.79 (1H, t, 9.28) | 70.22, d | - | H-11-OH, H-14 | - |
| 12 | 1.28 (1H, m) | 39.18, t | C-10, C-11, C-13 | H-11 | - |
|  | 1.08 (1H, m) |  | C-7, C-8 C-10, C-11, C-13 | H-4, H-9 | - |
| 13 | 3.32 (1H, m) | 67.67, d | - | H-12, H-13-OH | - |
| 14 | 1.22 (2H, m) | 39.41, t | C-10, C-11 | H-11 | - |
| 15 | 1.58 (1H, m) | 45.93, d | C-13, C-14, C-16, C-30 | H-16, H-30 | - |

| 16 | 3.55 (1H, td, 2.64, 9.12) | 75.20, d | C-15, C-18, C-30 | H-15, H-16-OH, H-17, | - |
| --- | --- | --- | --- | --- | --- |
| 17 | 5.54 (1H, dd, 8.72, 15.36) | 137.01, d | C-15, C-16, C-19 | H-16, H-18 | - |
| 18 | 6.07 (1H, dd, 9.92, 15.28) | 131.13, d | C-16, C-19, C-20 | H-17 | - |
| 19 | 6.18 (1H, m) | 133.30, d | C-17, C-18, C-20, C-21, C-22 | - | - |
| 20 | 6.18 (1H, m) | 133.61, d | C-17, C-18, C-21, C-22 | - | - |
| 21 | 6.27 (1H, m) | 131.92, d | C-22, C-23 | - | - |
| 22 | 6.27 (1H, m) | 132.40, d | C-21, C-23 | - | - |
| 23 | 6.21 (1H, m) | 131.69,d | C-19, C-20, C-22, C-26 | - | - |
| 24 | 6.21 (1H, m) | 133.64, d | C-19, C-20, C-22, C-26 | - | - |
| 25 | 6.11 (1H, m) | 129.70, d | C-20, C-23, C-24, C-27 | - | - |
| 26 | 5.91 (1H, dd, 6.48, 15.6) | 137.02, d | C-24, C-25, C-27, C-28 | H-27 | - |
| 27 | 2.36 (1H, m) | 39.93, d | C-25, C-26, C-28, C-31, C-32 | H-26, H-28, H-31 | - |
| 28 | 4.63 (1H, m) | 74.71, d | C-1,C-26, C-27, C-31, C-32 | H-27, H-32 | - |
| 29 | 0.80 (3H, t, 7.44) | 11.84, q | C-2, C-3 | H-3a, H-3b | - |
| 30 | 0.87 (3H, d, 6.80) | 10.70, q | C-13, C-15, C-16 | H-15 | - |
| 31 | 0.99 (3H, d, 6.88) | 16.10, q | C-26, C-27, C-28 | H-27 | - |
| 32 | 1.17 (3H, d, 6.24) | 19.51, q | C-27, C-28 | H-28 | - |
| 4-OH | 4.85 (1H, s) | - | C-4, C-5, C-7 | H-4 | H-6-OH, H-9 |
| 6-OH | 5.26 (1H, s) | - | C-6 | H-6 | H-4-OH, H-9 |
| 9-OH | 4.90 (1H, s) | - | C-9, C-10, C-12 | H-9 | H-9 |
| 11-OH | 4.96 (1H, s) | - | C-10, C-11,C-14 | H-11 | H-9 |
| 13-OH | 4.34 (1H, s) | - | C-13, C-14, C-15 | H-13 | H-9 |
| 16-OH | 4.77 (1H, s) | - | C-15, C-16 | H-16 | H-9 |

**Table S2.** Effects of compound 1 on *A. fumigatus* cell wall and cell membrane related genes.

| **Gene name** | **Log_2_ (FC)** | **Regulated** | **Description** |
| --- | --- | --- | --- |
| *Afu1g01730* | -1.26 | down | glycosyl hydrolase |
| *ENGL1* | -1.17 | down | endo-1,3-beta-glucanase |
| *Afu1g09270* | -2.47 | down | transmembrane glycoprotein |
| *ags3* | -1.12 | down | alpha-1,3-glucan synthase |
| *aspf9* | 1.71 | up | extracellular cell wall glucanase Crf1/allergen |
| *hmg1* | -1.15 | down | HMG-CoA reductase |
| *ags2* | -2.71 | down | alpha-1,3-glucan synthase |
| *cspA* | -2.23 | down | cell surface protein |
| *Afu4g00620* | -1.10 | down | cell wall glycosyl hydrolase Dfg5 |
| *eng5* | 1.21 | up | endo-1,3(4)-beta-glucanase, |
| *Afu5g00670* | 1.44 | up | beta-galactosidase |
| *eng4* | -1.51 | down | endo-1,3(4)-beta-glucanase |
| *rodF* | -3.36 | down | conserved hypothetical protein |
| *rodA* | -3.77 | down | conidial hydrophobin Hyp1/RodA |
| *gprP* | -1.01 | down | IZH family channel protein (Izh3) |
| *scw4* | -3.30 | down | cell wall glucanase |
| *Afu8g00650* | -4.07 | down | LPS glycosyltransferase |
| *Afu1g06910* | 1.38 | up | arabinogalactan endo-1,4-beta-galactosidase GalA |
| *Afu5g02130* | 2.32 | up | alpha-galactosidase |
| *Afu8g01130* | 1.10 | up | alpha-galactosidase C |
| *Afu8g00670* | -3.39 | down | integral membrane protein |
| *Afu6g04280* | -1.82 | down | integral membrane protein |
| *Afu2g17760* | -1.71 | down | integral membrane protein |
| *chi5* | 1.21 | up | class III chitinase |

**Table S3.** Effects of compound 1 on oxidative stress-related genes in *A. fumigatus*.

| **Gene name** | **Log_2_ (FC)** | **Regulated** | **Description** |
| --- | --- | --- | --- |
| *fgaOx1* | -3.33 | down | FAD binding oxidoreductase CpoX1 |
| *fgaDH* | -3.22 | down | short chain dehydrogenase/oxidoreductase CpoX2 |
| *fgaCat* | -3.14 | down | catalase Cat |
| *Afu4g01440* | 2.01 | up | glutathione S-transferase 2 |
| *Afu5g15070* | 1.22 | up | AhpC/TSA family thioredoxin peroxidase |
| *fgaOx3* | -2.73 | down | NADPH dehydrogenase Oye3 |
| *encD* | -2.55 | down | oxidoreductase, 2OG-Fe(II) oxygenase family |
| *Afu1g15610* | 1.07 | up | oxidoreductase, zinc-binding dehydrogenase family |
| *Afu5g02300* | 1.33 | up | peroxidase |
| *ppoA* | 2.16 | up | fatty acid oxygenase PpoA |

**Table S4.** Effects of compound 1 on genes related to transmembrane transporters of *A. fumigatus*.

| **Gene name** | **Log_2_ (FC)** | **Regulated** | **Description** |
| --- | --- | --- | --- |
| *Afu5g10430* | -2.24 | down | MFS multidrug transporter |
| *Afu4g06050* | -1.92 | down | MFS multidrug transporter |
| *Afu4g00570* | -1.89 | down | MFS multidrug transporter |
| *Afu4g11780* | -1.72 | down | MFS multidrug transporter |
| *Afu3g02060* | -1.08 | down | MFS multidrug transporter |
| *aflT* | 1.6127 | up | MFS toxin efflux pump |
| *gliA* | 4.8089 | up | MFS gliotoxin efflux transporter |
| *atrF* | -1.077 | down | ABC drug exporter |
| *sitT* | -1.912 | down | ABC multidrug transporter |
| *Afu4g01050* | -1.48 | down | ABC multidrug transporter |
| *Afu3g03670* | -2.24 | down | ABC multidrug transporter |


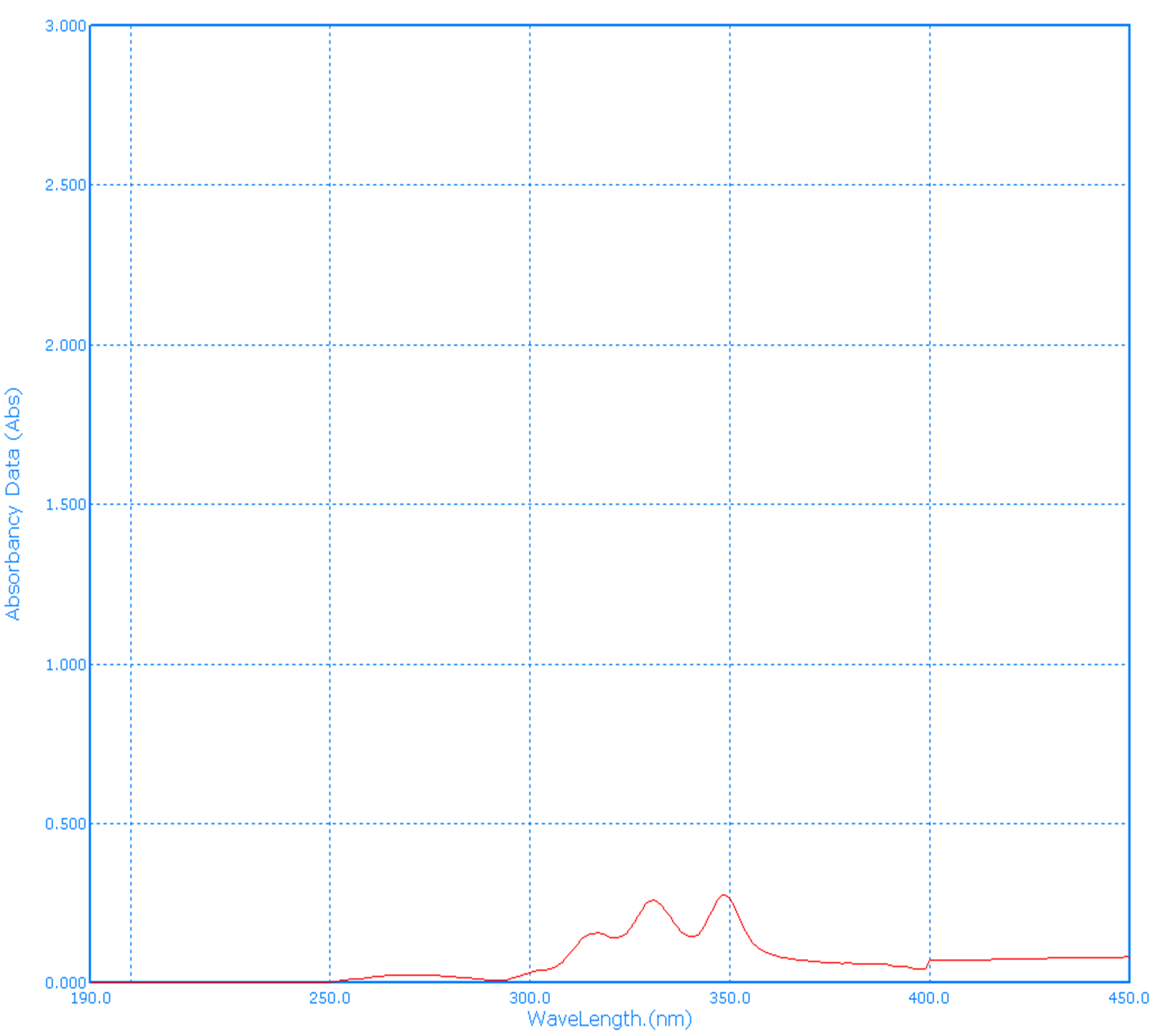
 **Figure S1.** UV peak diagram of compound **1**.


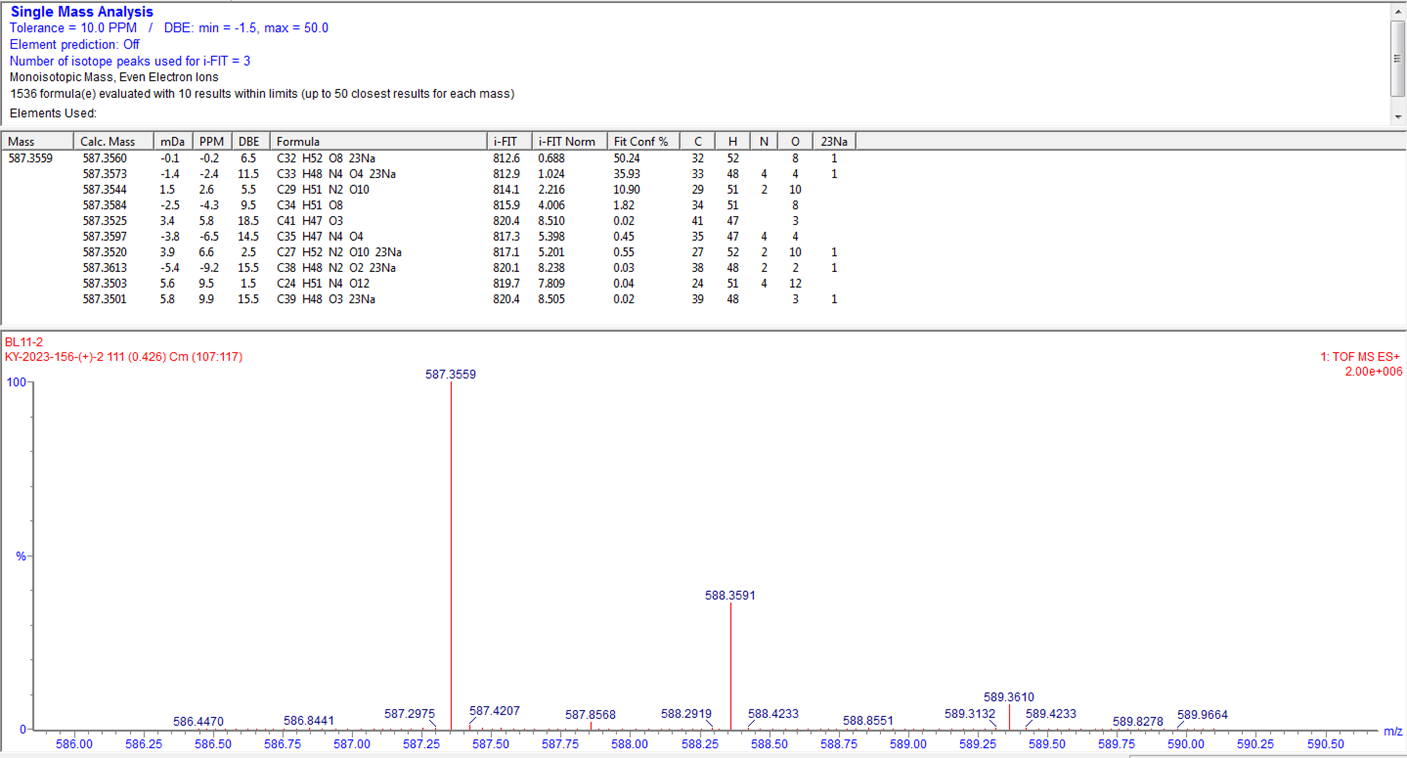


**Figure S2.** HR-ESI-MS spectrum of compound **1**.

**Figure S3.** ^13^C-NMR spectrum (800 MHz, MeOD) of compound **1**.

**Figure S4.** ^1^H-NMR(800 MHz, MeOD) spectrum of compound **1**.

**Figure S5.** HSQC spectrum of compound **1**.

**Figure S6.** COSY spectrum of compound **1**.

**Figure S7.** HMBC spectrum of compound **1**.

**
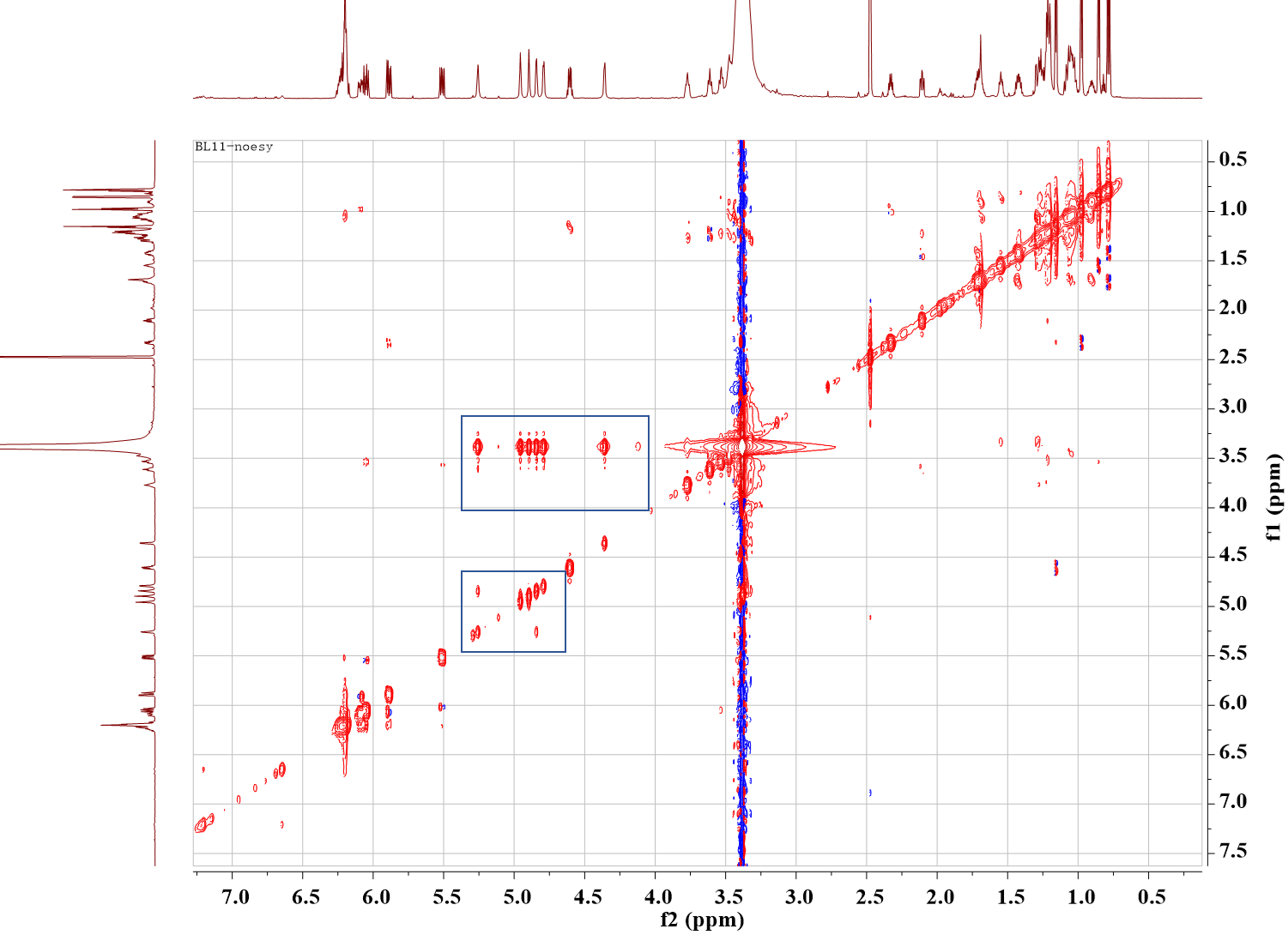
**

**Figure S8.** NOESY spectrum of compound **1**.
